# The incidence of acute kidney injury following cardiac arrest and cardiopulmonary resuscitation in a rat model

**DOI:** 10.1080/0886022X.2019.1596819

**Published:** 2019-04-24

**Authors:** Zhao-Yin Fu, Zhi-Jiang Wu, Jun-Hui Zheng, Tao Qin, Ye-Gui Yang, Meng-Hua Chen

**Affiliations:** Department of Critical Care Medicine, Second Affiliated Hospital of Guangxi Medical University, Nanning, P.R. China

**Keywords:** Acute kidney injury, rat, cardiac arrest, cardiopulmonary resuscitation, ischemia-reperfusion injury

## Abstract

**Objective**: In the current study, we investigated the incidence of acute kidney injury (AKI) induced by cardiac arrest (CA) and cardiopulmonary resuscitation (CPR) and whether such an AKI can recover spontaneously in rats.

**Methods:** We used transesophageal alternating current stimulation to establish 7 min of CA rat model followed by conventional CPR. The experimental rats were randomly divided into three groups (*n* = 20 per group) according to the different time points after restoration spontaneous circulation (ROSC): the ROSC 24 h, ROSC 48 h, and ROSC 72 h group. The diagnosis of rat AKI refers to the 2012 KDIGO adult AKI diagnostic criteria. The severity of AKI quantified by the serum creatinine (SCR), blood urea nitrogen (BUN) levels and histological features of renal tissue.

**Results:** The incidence rates of AKI in ROSC 24 h, ROSC 48 h, and ROSC 72 h group were 65%, 45%, and 42.9%. Moreover, the values of SCR and BUN were highest at ROSC 24 h, and then gradually decreased with the time of ROSC. The histological changes of the renal tissues such as glomerular collapse, renal tubular cell swelling, and inflammatory cell infiltration had also observed.

**Conclusion:** The incidence of AKI in rats was high after suffering from CA and CPR, but renal function improved with the prolongation of ROSC time, indicating the ability of the kidney to self-repair.

## Introduction

Acute kidney injury (AKI), previously called acute renal failure (ARF) [1], is caused by a variety of causes, including renal ischemia-reperfusion injury (IRI), and the Whole-body ischemia-reperfusion injury (WBIRI) was one of the most common causes of AKI after cardiac arrest (CA) [2]. Even if the first phase of resuscitation is successful after CA and cardiopulmonary resuscitation (CPR), all organs of the body suffer from WBIRI damage due to restoring spontaneous circulation (ROSC), resulting in multiple organ dysfunction syndromes (MODS) (i.e., post-resuscitation syndrome) [3]. Several studies have found that AKI was a common post-resuscitation syndrome that develops in approximately 30% of in-hospital cardiac arrest patients [4], While the incidence of AKI stage 3 occurred in out-of-hospital cardiac arrest (OHCA) patients was 48.3% [5].

At present, the subjects of ischemia-reperfusion injury after CA in animal experiments were mostly brain [6] and heart [7], but AKI was also associated with poor outcome [8]. Nevertheless, several studies have found that AKI may have an impact on neurological recovery [9,10]. Therefore, it is equally important to study AKI after CA and CPR, and the occurrence of AKI in CA rats have not been well-studied.

To investigate how many cases of AKI do occur following CA and CPR. We modified our previous rats’ model [11] to set the duration of cardiac arrest after transesophageal alternating current stimulation to 7 min and evaluated the renal function at ROSC 24 h, ROSC 48 h, and ROSC 72 h. Furthermore, we looked for the point in time at which peak kidney damage occurs and observed the incidence of AKI following CA and CPR.

## Materials and methods

### Animals

The animal study approved by the animal ethics committee of Guangxi Medical University. All animals received treatment in strict adherence to the National Research Council's 1996 Guidelines for the Care and Use of Laboratory Animals. Anesthetics were titrated in all surgical procedures to avoid unnecessary pain. Male Sprague-Dawley rats weighing 200–230 g purchased from the Experimental Animal Center of Guangxi Medical University (China, Nanning). All rats were housed in filter-top cages and given acidified water and sterilized food.

### Experimental cardiac arrest rat model

All rats fasted for 12 h but with free access to water before the operation. Experimental rats were intraperitoneally injected with sodium pentobarbital (45 *µ*g/g) for anesthesia, and an additional dose of 10 *µ*g/g was supplemented at hourly intervals. Standard Lead II ECG was used to monitor heart rhythm. A twenty-gauge catheter containing 5 IU/ml of sodium heparin saline was inserted into the right femoral vein for drug delivery, and another identical catheter was inserted into the right femoral artery for hemodynamic monitoring. Pressure transducers were connected to a four-channel physiological recorder (BL-420 E Biosystems, Chengdu Technology & Market Co. Ltd., China). After the 5-min baseline EEG and physiologic measurements, temperature probes were placed into the rectum. During the experiment, the rectal temperature was adjusted to approximately 37° C using a heat lamp or ice pack.

The rat cardiac arrest model was established according to a previously reported method [11]. Briefly, CA was induced by alternating current (12 V) from a stimulator (Chengdu Technology & Market Co. Ltd., China) through a pacing electrode placed in the esophagus, confirmed by a decrease in mean arterial pulse pressure <10 mmHg and by the appearance of asystole on the electrocardiograph. CPR was initiated seven min after induction of cardiac arrest with manual chest compressions. A metronome was employed in our experiment and its frequency was set as 180 per minute. We followed the metronome to conduct the manual chest compression, and effective ventilation (TV 8 mL/kg, respiration rate 40/min, and PEEP 0 cm H_2_O, oxygen concentration 100%) using a small animal ventilator with capacity control mode (DH-150, The Medical Instrument Department of Zhejiang University, China). After one min of CPR, one dose of epinephrine (0.4 *µ*g/g) was given through the left femoral vein catheter. When ROSC was clarified by ECG activity with visible systole [12] and mean arterial pressure (MAP)≥50 mmHg for ≥ 1 min, chest compressions were stopped. If ROSC is not achieved within 3 min of the onset of cardiopulmonary resuscitation, it is defined as a failure, and the animals is excluded from the study. After the return of spontaneous circulation (ROSC), rats were randomized into three groups (*n* = 20): the ROSC 24 h, ROSC 48 h and ROSC 72 h groups. The sham-operated rats only received the same experimental preparation without CA induction. The monitor and ventilator were deactivated after the rats resumed spontaneous breathing after ROSC 1 h. the rats were individually fed in cages with dry litter and placed in a quiet room with air conditioning adjusted temperature (room temperature 26˚C). In order to avoid unnecessary injury affecting the experimental results, each experimental group only took blood sample at observation time once from the right carotid artery, and then the rats were sacrificed to take bilateral kidneys. The right kidney tissue was used for Western Blot analysis, and the left kidney tissue was fixed with paraformaldehyde for histology assessment.

### Renal function analysis

The serum of the experimental rats was taken from the carotid artery at 24 h, 48 h, and 72 h after ROSC. The values of creatinine and urea nitrogen were monitored in the Department of Laboratory, the Second Affiliated Hospital of Guangxi Medical University. The diagnosis of rat AKI refers to the 2012 KDIGO adult AKI diagnostic criteria [13]

### Histologic assessment

The left kidney was fixed with paraformaldehyde and embedded in paraffin. Paraffin blocks were cut into 5 *µ*m thickness coronal sections and stained with hematoxylin and eosin (H&E).

### Statistical analysis

All data are expressed as mean ± SD. Statistical analysis software is SPSS 17.0 (SPSS, Inc., Chicago, IL, USA). Continuous variables between groups were compared using the Student t-test. One-way repeated-measures analysis of variance (ANOVA) or paired t-test was used to determine differences over time within groups, as appropriate.

## Results

### Baseline parameters for hemodynamics and CA/CPR

As Table 1 shows, there were no significantly different baseline parameters induction among all groups, including body weights (BW), mean arterial pressure (MAP) at ROSC 0 h, ROSC 1 hand before induction of cardiac arrest, the duration of transesophageal stimulation prior to CA (stimulation duration), and the duration of CPR prior to ROSC (CPR duration) (*p* > .05).

**Table 1.** Baseline parameters for hemodynamics and CA/CPR.

Group	*n*	BW	MAP (mmHg) before CA	MAP (mmHg) after ROSC 0 h	MAP (mmHg) after ROSC 1 hr	Stimulation duration (s)	CPR duration (s)
Sham	10	223.56 ± 25.31	93.1 ± 7.3	一	一	一	一
ROSC 24 h	20	220.12 ± 21.89	96.5 ± 8.8	82.5 ± 7.8	98.4 ± 5.3	60.16 ± 3.07	119.18 ± 49.30
ROSC 48 h	20	228.25 ± 23.26	97.3 ± 9.6	85.5 ± 3.9	97.6 ± 5.5	61.57 ± 2.20	117.64 ± 47.88
ROSC 72 h	20	226.37 ± 25.58	95.4 ± 9.4	84.1 ± 4.1	98.1 ± 4.1	59.61 ± 3.30	118.54 ± 48.63

BW: body weights; MAP: mean arterial pressure; CPR: cardiopulmonary resuscitation; ROSC: restoration spontaneous circulation.

### AKI parameters for each experimental group

As Table 2 shows, the CPR success rates of AKI in ROSC 24 h, ROSC 48 h, and ROSC 72 h group were 80%,85%, and 80%. Our results showed that the survival rates at ROSC 24 h were 93.8%, 88.2%, and 87.5%. Furthermore, we found that after 24 h of ROSC, no rat death was observed in each experimental group. The incidence of AKI in surviving rats was also highest in the ROSC24 group, at 80%, then showing a downward trend.

**Table 2.** AKI Parameters for each experimental group.

Group	*n*	CPR success rate *n* (%)	Survival at ROSC 24 h *n* (%)	Survival at ROSC 48 h *n* (%)	Survival at ROSC 72 h *n* (%)	Cases of AKI *n*	The incidence of AKI in surviving rats *n* (%)
Sham	10	一	一	一	一	一	一
ROSC 24 h	20	16/20 (80)	15/16（93.8）	一	一	13	13/15 (80)
ROSC 48 h	20	17/20 (85)	15/17（88.2）	15/15 (100)	一	9	9/15 (60)
ROSC 72 h	20	16/20 (80)	14/16（87.5）	14/14 (100)	14/14 (100)	6	6/14 (42.9)

AKI: acute kidney injury; CPR: cardiopulmonary resuscitation; ROSC: restoration spontaneous circulation.

### Indicators of kidney function

**Figure 1.**
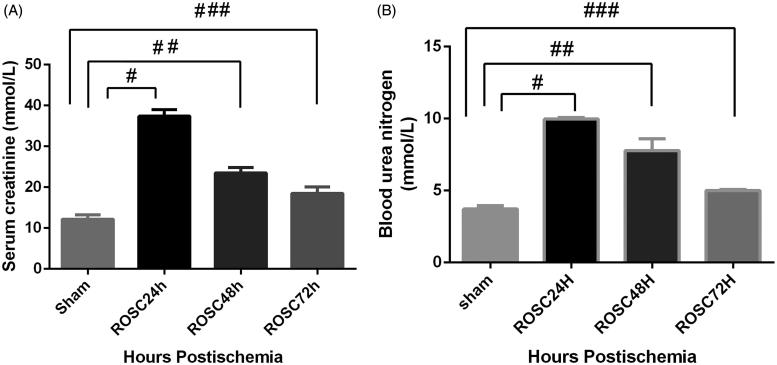
Changes in kidney function in rats after CA and CPR. Levels of (A) serum creatinine (SCR) and (B) blood urea nitrogen (BUN) were determined 24 h, 48 h, and 72 h after ROSC. SCR and BUN levels in the sham group were significantly lower than those in the model group. Data were expressed as mean ± SD #*p* < .01, ##*p* < .05 and ###*p* < .05. SCR: serum creatinine; BUN: blood urea nitrogen; CA: cardiac arrest; CPR: cardiopulmonary resuscitation.

We compared SCR and BUN as the renal function marker in all groups. The values of plasma SCR and BUN were significantly higher in each experimental group than in the sham-operated group. The rats subjected CA/CPR, including ROSC 24 h, ROSC 48 h and ROSC 72 h group, presented with progressively decreased SCR in a time-dependent manner. The ROSC 24 h group had the highest values of SCR. The same trend was also reflected in the results of the BUN (Figure 1(A,B)).

### Renal histopathology

**Figure 2.**
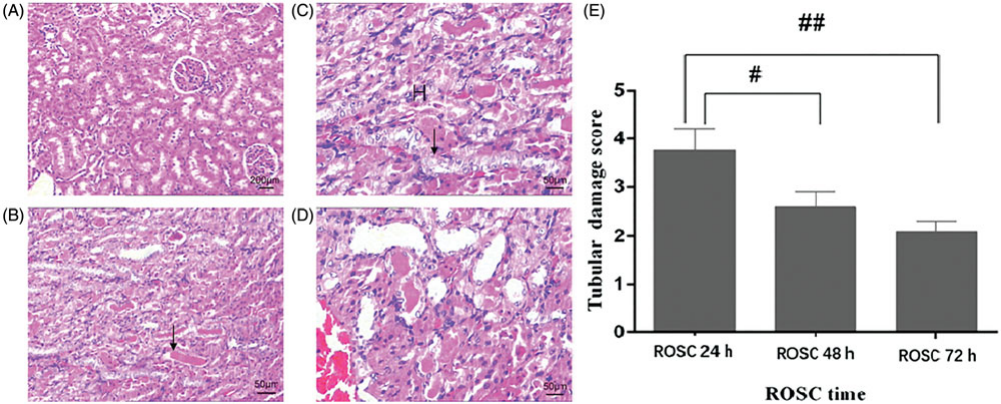
Light microscopy results. (A) sham-operated rat showed no tubular injury (magnification, x40). (B) After rat ROSC for 24 h, The kidney was extensively destroyed by excessive monocytes, increases in tubular necrosis, and tubular dilation (magnification, x100). (C,D) After rat ROSC for 48 h (magnification, x40) and 72 h (magnification, x100), there remained major tubular necrosis and cast formation. The arrows denote tubular necrosis and cast formation, ‘H’ denotes excessive monocytes. (E) The renal damage score was highest in the ROSC 24 h group compared with that in the ROSC 48 h and ROSC 72 h group. Results are expressed as mean ± SEM (*n* = 6 for each group) #*p* < .05, ##*p* < .01.

In the sham-operated group (Figure 2(A)), the renal tubules (including the proximal tubules and the distal tubules) and the renal corpuscles were seen. The structure of the renal tubule lumen was clear, and no obvious edema degeneration and accumulation of protein and cell debris in the official cavity were observed. After ROSC 24 h, 48 h, and 72 h, tubular epithelial cells are swollen, vacuoles in the cytoplasm, dilatation of the lumen, flat epithelium, various degeneration, and unequal exfoliation, cell debris in the lumen, basement membrane rupture, kidney Medullary congestion, red staining of protein in the tubules (Figure 2(B–D).

## Discussion

In the present study, we aimed to determine how many cased of acute kidney injury in rats subjected to CA/CPR. Our results showed that not all the rats experienced CA/CPR met the diagnostic criteria of AKI. Among the resuscitated rats, the incidence of AKI cases reached the maximum (80%) at 24 h after ROSC and then decreased over time. In addition, rats that underwent CA/CPR presented with increased SCR, BUN and more severe tubular damage compare with the sham group. The peak of these parameters appeared at 24 h after ROSC, especially the SCR with its 3-fold increase related to the sham group. Although the values of SCR and BUN were gradually decreased with time, their levels still fulfilled the requirement to be diagnosed with AKI according to the 2012 KDIGO criteria[13].

What factors may affect the occurrence of AKI after CA and CPR? The etiologies of AKI are multifactorial, but ischemia-reperfusion injury is one of the most important cause [14]. An essential hemodynamic feature during CA is systemic ischemia. It causes the immediate loss of renal perfusion, resulting in a nearly complete cessation of regional renal cortical blood flow (CBF) throughout CA. Chess compression and adrenaline application could restore MAP including regional renal CBF during and post CPR[15]. Besides, renal ischemic duration may be another important factor that contributed to the incidence of AKI. Regarding our model, we found that 10 min of WBIRI was sufficient to promote AKI in rats using a transesophageal AC-induced CA rat model modified from our previous study [11]. Interestingly, the ischemic time in our model required for AKI was similar to another study [16]. It is only one-third of the ischemic time required for the same kidney injury in the isolated renal artery clipping model, which needed at least 25 min to achieve the same injury [3,17]. The probable cause is that when the ischemia-reperfusion injury occurs after ROSC, the whole body simultaneously presented with damages in multiple organs [16], the harmful product released into the circulation, and the scavenging function aftershock can be used as an additional stimulus for AKI. In our current rat AKI model, the peak values of SCR and BUN in the present results were different from the previous studies. Multiple factors could contribute to this outcome, such as the renal ischemic time, the species of the experimental animal, the cause of CA induction, the duration of CA and the drug dosage.

Our CA model is closer to clinical data than others. Although isolation of the clamped renal artery is widely used in most kidney injury studies and the incidence of AKI is 100% [3,16], it does not mimic the clinical characters of human AKI. Because most clinical IRI cases occurred after whole-body IRI, and up to one hundred percent of the AKI incidence after CA and CPR was inconsistent with clinical data. An early clinical study had found that only 40.9% of patients who initially survived after CA and CPR usually developed AKI after resuscitation[18]. In addition, our results indicated that renal function gradually improved with ROSC time after AKI. This trend was similar to the murine and canine models with CA and without CPR performed for 10 min[16,19], and consistent with a clinical study on the timing of CA-induced AKI[5]. The reason may be related to the kidney's significant functional reservation and the strong self-repair function[20]. Moreover, we found that after ROSC, the 24-h survival rate of ROSC24h, ROSC48h, and ROSC72h groups was 93.8%, 88.2%, and 87.5%, respectively. The possible reason was when the body suffered from CA and CPR, even if the return of spontaneous circulation, the WBIRI resulting in multiple organ dysfunction syndromes (MODS). We suspect that rats may die from multiple organ failure.

We established a novel AKI experimental model after CA and CPR in rats. The mechanism of AKI after CA and CPR were performed in different animal experiments, including arterial or hilar clamping, nephron-sparing surgery, renal transplant and the induction of cardiac arrest was asphyxia or high potassium. In the previous study, the most severe renal impairment by systemic ischemia/reperfusion induced by asphyxia was reported at 24 h and 72 h after ROSC, respectively [3]. However, the incidence of adult asphyxia CA rarely appeared in clinical practice, and our previous research confirmed that hyperkalemia could ameliorate the brain I/R injury [21]. It is controversial whether there is a protective effect on renal ischemia-reperfusion injury in case of hyperkalemia. Therefore, unlike the conventional method of modeling AKI, we used the methods of transesophageal alternating current stimulation to conduct the rat CA model of 7 min followed by conventional CPR in order to avoid the confounding effects of surgical vascular manipulation, infection, hilar injury, venous congestion, cold renal ischemia, and provides us with a natural dual kidney warm ischemia model [20]. Moreover, the experimental subjects in CA model mostly were mice [15], but a previous study had shown that the degree of organ damage caused by ischemia and reperfusion injury after CA was different in mice and rats [12] Therefore, we used adult male rats as experimental subjects for the better simulation of AKI cases in adults after CA in the clinic.

This model has some limitations. First, it cannot fully mimic patients’ physiology status, the study results on this model must be interpreted according to the differences between species. In addition, our model had a cardiac arrest duration of 7 min with its survival rate of 80–85%, yet the patients’ survival rate was under 50% [22]. Even if the CPR was performed within 3 min of CA. Second, due to the small size of rat, the vascular intubation and chest compression is demanding. Third, we cannot collect the urine volume of rats after ROSC for now.

In conclusion, the CA/CRP rat model not only exhibited the biological markers and histological changes which were similar as the clinical cases of AKI, but also provided more important advantages compared with the other AKI models, including better simulation of clinical features of AKI after CA and CPR, easy specimen collection, low cost compared to large animals, and easy experimental operation.

## Ethics approval and consent to participate

The present study was approved by the Committee on the Ethics of Animal Experiments and Human Subject Research of the Guangxi Medical University.

## Disclosure statement

No potential conflict of interest was reported by the authors.

## Availability of data and materials

The analyzed datasets generated during the study are available from the corresponding author on reasonable request.
